# Possibilities for Cytoprotective Liver Therapy, Stable Gastric Pentadecapeptide BPC 157, and Standard Agents—A Narrative Review

**DOI:** 10.3390/ph19101559

**Published:** 2026-10-01

**Authors:** Marko Sever, Anita Zenko Sever, Robert Klicek, Lucija Kralj, Marta Popovic Dolic, Kresimir Luetic, Dinko Bekic, Zeljka Belosic Halle, Mario Sucic, Suncana Sikiric, Lidija Beketic Oreskovic, Antun Bacic, Sanja Strbe, Eva Staresinic, Sven Seiwerth, Predrag Sikiric, Alenka Boban Blagaic, Ivan Dobric, Mario Staresinic, Anita Skrtic

**Affiliations:** 1 Department of Pharmacology, School of Medicine, University of Zagreb, 10000 Zagreb, Croatiazeljkabelosic@gmail.com (Z.B.H.);; 2 Department of Surgery, School of Medicine, University of Zagreb, 10000 Zagreb, Croatia; rklicek@gmail.com (R.K.);; 3 Department of Pathology, School of Medicine, University of Zagreb, 10000 Zagreb, Croatialucijakralj13@gmail.com (L.K.);

**Keywords:** cytoprotection, hepatoprotection, cytoprotective agents, liver therapy, BPC 157, portal hypertension 42

## Abstract

Cytoprotection provides a conceptual framework for the preservation and restoration of tissue integrity under conditions of noxious injury. In liver disease, however, effective protection may require more than direct limitation of hepatocellular damage, encompassing endothelial function, microcirculation, vascular homeostasis, oxidative stress, inflammation, fibrosis, and portal hemodynamics. This review examines possibilities for cytoprotective liver therapy, with particular emphasis on stable gastric pentadecapeptide BPC 157 and comparison with established or experimentally investigated agents, including N-acetylcysteine, ursodeoxycholic acid, corticosteroids, resveratrol, L-arginine, curcumin, sildenafil, VEGF-related approaches, stem cell therapies, melatonin, simvastatin, prostacyclin analogues, and coenzyme Q10. Evidence is considered across representative experimental models of acetaminophen and carbon tetrachloride hepatotoxicity, bile duct ligation, and alcohol-induced liver injury. The diversity of effective agents indicates that liver injury involves multiple interconnected pathological mechanisms and that preservation of hepatocellular integrity may be achieved through different pharmacological pathways. BPC 157 is distinguished by evidence of beneficial effects across these major models and additional vascular and occlusion/occlusion-like syndromes. In particular, reported findings indicate prevention and reversal of experimentally induced portal hypertension, including prehepatic, intrahepatic, and posthepatic disturbances, together with attenuation of hepatic congestion, thrombosis, oxidative injury, and associated multiorgan dysfunction. These findings support the concept that cytoprotection may extend from hepatoprotection to restoration of vascular and tissue homeostasis. Collectively, the available preclinical evidence supports further investigation of integrated cytoprotective strategies for liver injury. Nevertheless, experimental evidence, however extensive, does not establish clinical efficacy, and appropriately designed clinical studies remain necessary to determine therapeutic applicability in human liver disease.

## 1. Introduction

The concept of cytoprotection [1,2,3,4,5,6,7,8,9] provides a rational framework for protective and restorative liver therapy. This should particularly consider those therapies involving the stable gastric pentadecapeptide BPC 157 (for review, see, i.e., [10,11]) and standard agents also thought to be cytoprotective [12,13,14,15,16,17,18,19,20,21,22,23,24,25,26,27,28,29]. These agents have been reported to exert cytoprotective or hepatoprotective effects and have consequently been investigated in experimental models of liver injury [12,13,14,15,16,17,18,19,20,21,22,23,24,25,26,27,28,29]. Accordingly, we selected N-acetylcysteine [12,13], ursodeoxycholic acid [14], corticosteroids [15,16,17,18], resveratrol [19,20], L-arginine [21], curcumin [22], sildenafil [23], experimental VEGF gene delivery [24], stem cell therapies associated with VEGF activation [25], melatonin [26], simvastatin [27], prostacyclin analogues [28], and coenzyme Q10 [29] as comparative agents when considering the cytoprotective efficacy of BPC 157. Comparing BPC 157 with other experimentally investigated hepatoprotective agents matters because liver injury involves multiple, interconnected mechanisms, including oxidative stress, inflammation, endothelial dysfunction, disturbed microcirculation, impaired tissue repair, and, in chronic settings, fibrogenesis. Accordingly, a broad range of pharmacological and regenerative approaches has been evaluated in experimental liver lesion models (i.e., acetaminophen, carbon tetrachloride (CCl_4_), bile duct ligation, alcohol-induced liver lesions). These agents are not mechanistically equivalent and therefore should not be regarded as interchangeable standard comparators. Rather, they represent distinct therapeutic strategies, including antioxidant and glutathione-dependent protection, bile-acid modulation, anti-inflammatory treatment, NO-dependent vascular modulation, angiogenesis, endothelial and microcirculatory protection, mitochondrial preservation, and tissue regeneration (Table 1). Their inclusion was agent (i.e., N-acetylcysteine) combined with the model (i.e., acetaminophen), using https://pubmed.ncbi.nlm.nih.gov, including studies till August 2026, excluding non-English studies, providing that this article was designed as a narrative review rather than a systematic or scoping review. This provides a useful framework with illustrative manuscripts for evaluating whether the effects of BPC 157 remain restricted to a particular pathogenic pathway or encompass several components of the liver injury response. Although this approach may not completely avoid the problem of proper balance, this broader comparison is of particular interest because BPC 157 has demonstrated protective effects in different experimental models of liver injury, including toxic, biliary/vascular, radiation-induced, and secondary ischemia-reperfusion-associated liver injury [10,11].

This would be the focus of the present review. Beyond summarizing current evidence (at least as may be seen from mentioned classic models (i.e., acetaminophen, CCl_4_, bile duct ligation, alcohol-induced liver lesions)), in the cytoprotection concept, the central idea is that protection is not specific to one particular organ or one particular noxious agent [1]. Rather, a cytoprotective agent enhances tissue resistance and promotes preservation of cellular integrity against a broad range of injuries [1]. This specifically also includes the maintenance and preservation of endothelial function [4,5,6,7,8,9]. Thus, this liver review aims to bridge the gap in the concept’s full realization in therapy and the gap between experimental animal studies and future clinical applications by reconsidering liver cytoprotection from the realizable perspective of the original concept of cytoprotection. A general advantage of experimental animal studies that should be implemented in translational therapeutic development is the degree of mechanistic and methodological control that cannot be achieved in human studies, meaning a clear pathway: basic-study advantage → implementation → therapeutic development.

Consequently, these basic studies provide a higher level of confidence regarding causal mechanisms, thereby offering a robust scientific foundation for resolving current limitations in the clinical investigation of human liver disease through the application of the cytoprotection concept [30,31]. This may be particularly challenging in conditions where injury progression becomes uncontrolled or maladaptively regulated [1]. Nevertheless, the pleiotropic issue of liver healing and cytoprotection (i.e., specifically, maintaining and/or reestablishing hepatocellular, endothelial, and tissue integrity) remains a central challenge in hepatology and pharmacology [32,33,34,35].

Notably, as an additional way out, BPC 157 administration was an effective protective/recovering therapy against the mentioned classic liver lesion models (i.e., acetaminophen [36], CCl_4_ [37], bile duct ligation [37,38], alcohol-induced liver lesions [39,40]). Moreover, this includes the recovery of others, such as reversing Budd–Chiari syndrome [41], Pringle maneuver ischemia/reperfusion [42], and other occlusion/occlusion-like syndromes, where severe hepatic venous congestion and liver injury occurred as part of a systemic venous occlusion syndrome [43,44,45,46,47,48,49,50,51,52,53,54,55,56,57,58]. Likewise, there is the counteraction of irradiation-induced liver lesions [59] and the counteraction of non-steroidal-anti-inflammatory-drug-induced liver lesions [60,61,62,63,64,65]. Such an effect also includes broiler chicken [66]. As a consistent part of its effectiveness, it counteracted portal hypertension, preventing development and counteracting already established, equally prehepatic, intrahepatic, and posthepatic portal hypertension [38,39,40,41,42,43,44,45,46,47,48,49,50,51,52,53,54,55,56,57]. This highlights evidence that the peptide affects both parenchymal injury and vascular disturbances/circulatory dysfunction. Portal hypertension represents a major consequence of chronic liver disease and reflects not only fibrosis but also endothelial dysfunction, disturbed hepatic microcirculation, altered NO signaling, thrombosis, angiogenesis, and oxidative stress [67,68,69,70]. Therefore, therapeutic approaches that improve portal hemodynamics may represent an important extension of the cytoprotection concept.

With the stable gastric pentadecapeptide BPC 157 versus/along with standard agents, commonly combined with cytoprotection, we considered the applicable effectiveness of these agents throughout the mentioned models of liver lesions commonly used in basic studies. Likewise, their capability to counteract various extrahepatic lesions through these liver lesion models (cytoprotective agent ≈ pleiotropic beneficial effects; cytoprotection → organoprotection).

To emphasize, in the cytoprotection concept, stable gastric pentadecapeptide BPC 157 may have a special role as when it is native and stable in human gastric juice (more than 24 h), it acts as a cytoprotection mediator [10,11,30,31]. Thereby, its beneficial pleiotropic cytoprotective and hepatoprotective effects, in particular, occurred regardless of the route of application. Therefore, BPC 157 behaves unlike other peptides. However, since it is always applied alone without carrier, BPC 157 has a clear effect attribution to peptide itself. Contrarily, with standard peptides, delivery problems are quite common, such as use of the carriers, and consequently, activity attribution problems related to use of peptide + carrier(s) complexes (additionally, other peptides are easily destroyed in gastric juice) [10,11,30,31]. As an advantage, toxicological studies have demonstrated an exceptionally favorable safety profile, with no lethal dose achieved (LD_1_ not reached) even at doses of 2 g/kg i.v. or i.g. in mice (for more details see, i.e., [10]). Human data remain limited but encouraging, although these findings are not for clinical efficacy and/or safety data for liver disease. BPC 157 was found to be effective and well tolerated in phase II clinical trials in ulcerative colitis [71,72] (for more details on favorable early human tolerability, see i.e., [10]), and subsequently in small clinical studies addressing knee pain and interstitial cystitis, all without reported adverse effects [71,72,73,74]. These findings are supported by toxicological evaluations carried out by other groups and patient reports [75,76,77,78,79], consistent with previous elaboration [10,11,30,31].

At the general level and in the cytoprotection as a unified-system-level model, the search may have particular problems in specifying specific mechanistic points. For instance, Robert’s essential concept of pushing physiology back toward normality, at least in theory, requires bidirectional action of a cytoprotective agent to restore physiological equilibrium. Thereby, while BPC 157 was implicated in interacting with various molecular pathways [80,81,82,83,84,85,86,87,88,89,90], in the healing course induced by BPC 157, particular emphasis was placed on the mechanisms occurring throughout, which may be especially relevant for the preservation of liver structure and function during toxic, vascular, inflammatory, and multiorgan injury (for review, see, i.e., [10]). Specifically implicated were its rapid activation of the collateral vascular pathways and its ability to act as a safety key, a dual response, context- and tissue-dependent, modulating and resolving angiogenesis, vascular endothelial growth factor (VEGF) activity, the early growth response-1 (egr-1) gene, and stimulation of nitric oxide (NO) pathways and endothelial NO synthase (eNOS) [10]. There, BPC 157 may act as a modulator of NO-system function, including its own NO-release, particular interactions with NO-agents, both NO-blocker and NO-substrate, and particular interaction with NO-molecular pathways. Namely, it counteracts adverse effects of NO-blockade (L-NAME-hypertension, prothrombotic effect) or NO-overstimulation (L-arginine-hypotension, anticoagulant effect) [10]. This can preserve endothelial integrity and hepatic microcirculation. Regularly, BPC 157 exhibits counteraction of excessive free radical formation and oxidative stress, whatever its relation to the effect on NO-level (↑NO =↓MDA; ↓NO =↓MDA). This was considered as a particular therapy safety key [10], unlike simply acting as a NO agonist or antagonist. Rather, experimental findings suggest context-dependent normalization of disturbed NO signaling, with the direction of the response depending on the pre-existing state of the NO system [10]. For the particular rescuing vascular effect, the rapid activation of collateral rescuing pathways, and advanced tissue healing (azygos vein direct blood flow delivery), there is a confirmatory point from Fourier-transform infrared spectroscopy (FTIR) studies [51,91]. These support the previously observed rapid functional vascular effects of BPC 157 by measurable rapid changes in the molecular organization of vascular tissue [51,91]. Similar notations have been observed also in human internal mammary artery [92]. Additionally, a similar dual (modulatory) action (i.e., either hypertension or hypotension reversed toward normal blood pressure) seems to be a shared effect, as BPC 157 counteracts similar disturbances also with dopamine, serotonin, glutamate, GABA, adrenalin/noradrenalin, and acetylcholine, as seen with NO-system disturbances [93]. This neurotransmitter-like activity [93] envisaged in the implementation of the cytoprotection effects [93] specifically includes counteraction of those disturbances related to their receptors, both blockade and over-activity, destruction, depletion, tolerance, sensitization, and channel disturbances counteraction [93]. However, how a single pentadecapeptide senses the pre-existing tissue state to dictate an up- or down-regulation to restore system homeostasis remains undermined 182 (as it is still with Robert’s “pushing physiology back toward normality” [1,2,3,4,5,6,7,8,9]). However, for NO pathways, the evidence of particular interaction of BPC 157 with signaling pathways controlling vasomotor tone [82,83,84] (VEGFR2-Akt-eNOS and Src-Caveolin-1-eNOS) may be an indicator how to restore NO-system homeostasis, which may represent a central mechanism underlying its wide-ranging therapeutic potential.

Likewise, as reviewed [10,11,30,31] and demonstrated [38] also in the liver, this goes with bile duct ligation cirrhosis counteraction, pathological neovascularization inhibition, portal hypertension counteraction, and counteraction of hepatic fibrosis/cirrhosis. Thus, BPC 157 may promote resolution of the otherwise persistent and maladaptively sustained wound-healing response (i.e., reduced α-SMA-positive hepatic stellate-cell change, reduced hepatic IL-6, TNF-α, and IL-1β [38]) underlying hepatic fibrosis and cirrhosis [94,95,96,97,98] (see Section 2.4). Consistently, on the other hand, in the context of BPC 157 producing wound healing as context-dependent and tissue-dependent [10,11,30,31], BPC 157 inhibited corneal neovascularization (along with corneal ulcer healing and maintained transparency) [99] while advanced tissue healing angiogenesis occurred, i.e., in tendon, muscle, ligament, and other tissues [100]. Since BPC 157 was always given alone, without carriers (as it was not with other standard peptide delivery), these were indicative of combined function and tissue integrity recovery [100]. All of these effects may represent essential mechanisms underlying cytoprotection, hepatoprotection, and organoprotection, thereby contributing to the resolution of complex liver injury and restoration of tissue homeostasis. On the other hand, there is a remark on relevance: the central object of the review—BPC 157—remains a drug with predominantly preclinical status and an ambiguous regulatory history (references to FDA Pharmacy Compounding Advisory Committee meetings). From this perspective, this review will analyze the basic background for the given agents, animal models as a highlight for clinical efficacy, along with BPC 157 and the chosen standard agents (Table 1). Strong and reproducible basic research provides increasingly compelling evidence and justifies clinical development, but it cannot, by itself, establish clinical efficacy in humans. Therefore, we assume cytoprotection as a fundamentally tissue-homeostatic phenomenon rather than a drug class. Thus, the question is whether different liver injury models could reveal common protective mechanisms that extend from hepatoprotection to vascular and multiorgan protection. In other words, the stronger and more comprehensive the preclinical evidence (Table 1), the higher the expectation of clinical success, but the expectation is not the same as proof.

**Table 1:** Representative Experimental Liver Injury Models Used for Evaluation of Cytoprotective and Hepatoprotective Agents (NAFLD = Nonalcoholic Fatty Liver Disease; NASH = Nonalcoholic Steatohepatitis).

Agent	Representative Experimental Liver Injury Models	Principal Mechanisms Investigated	Representative References
**BPC 157**	Acetaminophen hepatotoxicity; diclofenac-induced liver injury; alcohol-induced injury; CCl_4_ injury; bile duct ligation; portal hypertension; Budd–Chiari-like syndrome; hepatic encephalopathy/multiorgan failure	Endothelial protection; NO-system modulation; collateral vessel recruitment; angiogenesis modulation; antioxidant and anti-inflammatory effects; cytoprotection/organoprotection	[36,37,38,39,40,41,42,43,44,45,46,47,48,49,50,51,52,53,54,55,56,57,58,59,61,62,63,64,65]
**N-acetylcysteine**	Acetaminophen toxicity; CCl_4_ injury; ischemia-reperfusion; alcohol-induced injury; bile duct ligation; lipopolysaccharide- induced injury	Glutathione replenishment; ROS scavenging; mitochondrial protection; detoxification of reactive metabolites	[12,13,101,102,103]
**Ursodeoxycholic acid**	Bile duct ligation; α-naphthylisothiocyanate-induced cholestasis; acetaminophen; CCl_4_ injury; alcohol-induced injury; estrogen-induced cholestasis; NASH; ischemia-reperfusion	Membrane stabilization; anti-apoptotic activity; bile acid detoxification; anti-inflammatory effects	[14,104,105,106]
**Corticosteroids**	Autoimmune hepatitis; ConA hepatitis; acetaminophen; CCl_4_ injury; bile duct ligation; lipopolysaccharide-induced injury; alcohol-induced injury	Cytokine suppression; immunomodulation; inhibition of inflammatory infiltration	[15,16,17,18,107]
**Resveratrol**	NAFLD; alcohol injury; CCl_4_ fibrosis; ischemia-reperfusion; bile duct ligation; acetaminophen toxicity	Antioxidant effects; SIRT1 activation; anti-inflammatory activity; mitochondrial preservation	[19,108,109]
**Curcumin**	CCl_4_ injury; alcohol injury; NAFLD/NASH; acetaminophen; bile duct ligation; thioacetamide fibrosis; ischemia-reperfusion	NF-κB inhibition; antioxidant activity; anti-fibrotic effects	[22,110,111]
**L-arginine**	Ischemia-reperfusion; portal hypertension; endotoxin injury; CCl_4_ injury; alcohol- induced injury	NO generation; microcirculatory improvement; endothelial protection	[21,61,62,112]
**Sildenafil**	Portal hypertension; ischemia-reperfusion; acetaminophen; CCl_4_ injury; bile duct ligation; fibrosis; cholestatic injury	PDE5 inhibition; NO-cGMP activation; portal pressure reduction	[23,113,114,115]
**VEGF Gene Delivery**	Partial hepatectomy; ischemia-reperfusion; fibrosis/cirrhosis; CCl_4_ injury; bile duct ligation; hepatic artery ligation	Angiogenesis; endothelial repair; regeneration	[24,116,117]
**Stem Cells with VEGF Activation**	CCl_4_ fibrosis; acute liver failure; ischemia-reperfusion; bile duct ligation	VEGF-mediated angiogenesis; regenerative signaling; endothelial repair	[25,118]
**Melatonin**	Ischemia-reperfusion; acetaminophen toxicity; bile duct ligation; CCl_4_ injury; alcoholic injury; NAFLD/NASH	Antioxidant activity; mitochondrial stabilization; anti-apoptotic effects	[26,119,120,121,122,123]
**Simvastatin**	Portal hypertension; cirrhosis; acetaminophen; CCl_4_ injury; bile duct ligation; ischemia-reperfusion	eNOS activation; endothelial protection; reduction of intrahepatic resistance	[27,124,125,126,127]
**Prostacyclin** **analogues**	Ischemia-reperfusion; portal hypertension; acetaminophen; CCl_4_ injury; bile duct ligation; liver transplantation; endotoxin injury	Vasodilation; platelet inhibition; endothelial stabilization	[28,128,129,130,131]
**Coenzyme Q10**	Acetaminophen toxicity; CCl_4_ injury; alcoholic liver disease; NAFLD/NASH; ischemia-reperfusion	Mitochondrial preservation; ATP maintenance; antioxidant activity	[29,132,133]

Unfortunately, this basic evidence has resulted in only limited therapeutic application. Likely, this could be far below the evidence that the cytoprotection concept itself extends across multiple mechanisms of liver injury and repair. Therefore, the possibilities for more cytoprotective liver therapy, tissue- and context-dependent, using the stable gastric pentadecapeptide BPC 157 and standard agents will be analyzed.

## 2. General Significance of Liver Lesion Models (Acetaminophen, CCl_4_, Bile Duct Ligation, Alcohol-Induced Liver Lesions)

As mentioned, in general terms, the appropriate interpretation of animal (rodent) liver lesions may considerably contribute to and improve clinical application in human liver diseases. This means not ignoring but emphasizing the internal strengths of animal models. Notably, in general, animal studies already have inherent controls impossible in humans: genetic homogeneity, controlled environment, and standardized injury models. Therefore, in liver lesion models in particular, the principle may be especially important, as they approach several key mechanisms involved in human liver disease. Thus, depending on the model used, the counteraction of the particular liver lesions in animals by the agent application may have particular significance. Not only in theory but quite specifically, this would mean attribution of mechanistically counteracting the various indicated patterns in basic research, including oxidative stress, mitochondrial dysfunction, endothelial injury, inflammatory activation, microcirculatory disturbances, and hepatocellular necrosis. Providing that in the animal experiment, the level of control largely exceeds that possible in humans, these may be highly predictive of human disease outcomes. Notably, as no experimental model fully reproduces the complexity of human liver disease, this means the necessity of combining models.

Therefore, within the cytoprotection concept, an agent may, in theory, be effective against all, or a broad range of, noxious events, thereby exhibiting a broad cytoprotective effect and indicating the presence of shared cytoprotective mechanisms. Alternatively, its effectiveness may occur against only some noxious events, indicating effects more specifically related to those particular toxic or pathological pathways.

### 2.1. Acetaminophen (Paracetamol) Hepatotoxicity

Acetaminophen liver injury remains the leading cause of acute liver failure in many Western countries [134,135,136]. Following overdose, the normal pathways of glucuronidation and sulfation become saturated, leading to increased cytochrome P450-mediated formation of the highly reactive metabolite *N*-acetyl-*p*-benzoquinone imine (NAPQI). Excessive NAPQI depletes hepatic glutathione stores, covalently binds cellular proteins, induces mitochondrial oxidative stress, impairs ATP production, activates c-Jun N-terminal kinase (JNK) signaling, and ultimately causes centrilobular hepatocyte necrosis. Beyond direct hepatocellular injury, acetaminophen toxicity is increasingly recognized as involving mitochondrial dysfunction, oxidative and nitrosative stress, sterile inflammation, endothelial injury, and microcirculatory disturbances, making it a particularly suitable model for evaluating cytoprotective and hepatoprotective interventions [134,135,136]. Consequently, acetaminophen hepatotoxicity has become a cornerstone model for investigating antioxidant, mitochondrial-protective, anti-inflammatory, endothelial-protective, and organoprotective therapies. There, the counteractions occur with many agents, N-acetylcysteine [137,138,139,140,141,142,143,144,145,146], resveratrol [147,148,149,150], curcumin [151,152,153,154,155,156], melatonin [157,158,159], coenzyme Q10 [160,161,162,163], corticosteroids [164], and sildenafil [165].

Notably, transplanted hepatocytes rescue mice in acetaminophen-induced acute liver failure [166].

Prostacyclin analog effects were shown in hamster hepatocytes and a rat epithelial cell line [167,168].

Simvastatin has a particular effect. In lower doses, it restored the decrease in malonyldialdehyde and myeloperoxidase levels, superoxide dismutase, catalase, glutathione, and glutathione peroxidase activities induced by acetaminophen hepatotoxicity but had no effect on liver regeneration. Moreover, high doses could aggravate acetaminophen-induced liver injury [169].

Ursodeoxycholic acid is a drug approved by the Food and Drug Administration of the USA to treat primary biliary cholangitis (PBC) [170]. Ursodeoxycholic acid was claimed to be not effective in in vivo and in vitro toxic liver injury in rats [171].

Surprisingly, L-arginine itself was not investigated in paracetamol liver studies. However, this should be seen with the likely essential role of the NO-system in the liver, and particularly, the dual role of the NO-system in acetaminophen liver lesions. It was claimed that NO derived from iNOS contributes to acetaminophen-induced parenchymal cell injury and hepatic microcirculatory disturbances (L-NIL exerts preventive effects), while NO derived from constitutive isoforms of NOS exerts a protective role in liver microcirculation against acetaminophen intoxication and thereby minimizes liver injury [172]. To this point, unlike L-NIL-induced preventive effects, NOS-blockers provide a shared aggravating effect [172,173], and NO-donors, NCX-1000, V-PYRRO/NO, a shared beneficial effect [174,175,176,177,178,179].

Thus, acetaminophen hepatotoxicity as a distinctive cytoprotective background should emphasize the cytoprotection commonality of particular agents. Therefore, it seems that the sharing class specifically related to the acetaminophen background described above [134,135,136] includes N-acetylcysteine, resveratrol, curcumin, melatonin, coenzyme Q10, corticosteroids, and sildenafil. Notably, their effectiveness includes pretreatment, cotreatment, posttreatment, and both pretreatment and posttreatment regimens. N-acetylcysteine includes pretreatment [138], cotreatment [141], posttreatment [139,140,142,143,144,146], and pretreatment and posttreatment [137]. Likewise, resveratrol includes pretreatment [149,150] as well as cotreatment [148] and posttreatment [147]. Curcumin includes pretreatment [153,154,155,156], cotreatment [152], and posttreatment [151], melatonin pretreatment [157,158], and pretreatment and posttreatment [159]. Coenzyme Q10 includes only posttreatment [140,160,161,162,163], sildenafil posttreatment [180], while corticosteroids include pretreatment [164]. The others, i.e., prostacyclin analogs, simvastatin, ursodeoxycholic acid, and experimental delivery of VEGF genes, remain outside of the acetaminophen loop.

On the other hand, acetaminophen hepatotoxicity as a distinctive cytoprotective background may be markedly wider. This should be evident in the large number of other agents from distinct classes that counteract acetaminophen hepatotoxicity in animal models.

A more suitable presentation would be in Table 2.

Thus, these (i.e., acetaminophen/applied beneficial agents as a cytoprotection background) illustrate that cytoprotection is not a distinct pharmacological class but rather a shared therapeutic property across diverse drug groups. This means a wide cytoprotection concept and different particular pathways leading to the same outcome (cell protection). Bioactivation inhibition [182,184,185], cAMP signaling [187], NO/cGMP signaling [186], regenerative therapy [192], and antioxidant therapies [152,180,195,200] are all under the paracetamol umbrella.

Moreover, these further points (i.e., counteraction of the paracetamol as a cytoprotection background by the same (cytoprotection) agent application transmitted to other organ therapy (cytoprotection→organoprotection)) show that the agents that protect against acetaminophen-induced liver injury have also demonstrated protection of extrahepatic organs (kidney, testis, bone marrow, brain, lung, etc.) (Table 3).

Thus, acetaminophen studies are not limited to liver injury alone. Historically, acetaminophen hepatotoxicity was primarily investigated as a model of acute liver failure. On the other hand, there is a widely recognized severe acetaminophen intoxication as a multiorgan disorder involving the kidney, brain, cardiovascular system, lungs, coagulation system, endothelium, and systemic inflammatory responses. Vice versa, counteraction of acetaminophen occurs as a cytoprotection background transmitted to other organs therapeutically by the same (cytoprotective) agent application. As a follow-up, there is multiorgan protection, simultaneous protection of liver and extrahepatic organs that quite commonly occurs with the agents that protect against acetaminophen-induced liver injury.

On the other hand, these points seem not to be appreciated in the clinic. So far, there is only one established clinical antidote for acetaminophen poisoning [135]. Additionally, because of the narrow therapeutic window for acute liver injury and the side effects of N-acetylcysteine, its clinical application has great limitations [204].

#### BPC 157/Acetaminophen

In contrast, this would not occur with BPC 157 (10 µg-10 ng/kg b.w. as a regular regimen, also in acetaminophen studies) [36]; toxicological studies have demonstrated an exceptionally favorable safety profile, with no lethal dose achieved (LD_1_ not reached), even at doses of 2 g/kg i.v. or i.g. in mice (for more details, see review [10]). Furthermore, the BPC 157/paracetamol relationship should be perceived in light of the presented findings with all of the mentioned agents. Notably, acetaminophen toxicity is usually induced experimentally at doses of 300–1000 mg/kg in mice and 0.5–3 g/kg in rats (see, Table 2). However, this range is largely exceeded. BPC 157 studies used 5 g/kg intraperitoneal acetaminophen, representing an exceptionally severe intoxication model with marked multiorgan involvement [36]. Thus, it can correlate with extreme suicidal overdose, fulminant hepatic failure, severe toxic encephalopathy, and impending multiorgan collapse. BPC 157 therapy was administered either immediately after acetaminophen or 3 h later at 10 µg/kg, 10 ng/kg, even 10 pg/kg, given intraperitoneally, or intragastrically. The peptide reportedly attenuated both hepatic and encephalopathic manifestations.

This may be relevant (two routes of administration in the same model) when discussing the robustness and translational significance of the observed therapeutic effects, even considering rats/human dose ratio. Thus, in rats, BPC 157 also neutralized the adverse effects of acetaminophen overdose in the liver (hepatomegaly, fatty liver and necrosis, pronounced elevation of liver enzymes and hyperammonemia). This occurs along with counteraction of the convulsions and severe brain injuries [36]. The rat data demonstrate a pharmacologically active effect in doses that, by conventional body-surface-area scaling, correspond to very small human-equivalent doses, including approximately 1.6 ng/kg for the 10 ng/kg rat dose. This provides a notable translational signal, although it cannot establish an effective or safe human dose, particularly because the experimental paracetamol exposure was an extreme overdose and the pharmacokinetics and exposure-response relationship of BPC 157 in humans remain insufficiently defined. However, although the rat paracetamol dose represents an extreme exposure and cannot be directly extrapolated to human poisoning, conventional body-surface-area scaling places the 5 g/kg rat exposure at approximately 0.81 g/kg human-equivalent dose, emphasizing the severity of the experimental challenge.

This should be seen along with the essential background of acetaminophen hepatotoxicity. As mentioned earlier [134,135,136], the reaction of superoxide with NO and the subsequent generation of peroxynitrite within the mitochondria are critical mediators of acetaminophen-induced mitochondrial dysfunction [205,206,207,208]. In this, BPC 157 may have a particular modulatory role. As emphasized before, BPC 157 may induce release of NO on its own and counteract adverse effects of NO-blockade (L-NAME-induced hypertension and procoagulant effect) and adverse effects of NO-over-activity (i.e., L-arginine-induced hypertension, and anticoagulant effect) (for review, see [10]). It may interact with many molecular pathways, especially with NO-molecular pathways [80,81,82,83,84,85,86,87,88,89,90]. BPC 157 exhibits a distinctive effect on the NO-level (increase vs. decrease), always combined with the counteraction of free radical formation, and, in mice and rats, BPC 157 therapy counteracts all disturbances (for review see [10]), in particular that of acetaminophen [36], that may be related to uncontrolled NO-system functioning. Therefore, acetaminophen intoxication may serve as an example where BPC 157 appears to combine several cytoprotective domains that are otherwise distributed among separate drug classes.

### 2.2. CCl_4_-Induced Liver Injury

CCl_4_-induced liver injury is one of the most widely used and best-characterized experimental models for studying acute and chronic liver disease. Following hepatic metabolism by cytochrome P450 enzymes, particularly CYP2E1, CCl_4_ is converted into highly reactive trichloromethyl (•CCl3) and trichloromethyl peroxy (•OOCCl3) radicals. These reactive intermediates initiate lipid peroxidation, membrane damage, mitochondrial dysfunction, oxidative stress, calcium dysregulation, inflammatory activation, and hepatocyte necrosis. Acute administration typically produces centrilobular necrosis and marked elevations of serum transaminases, whereas repeated administration leads to chronic inflammation, activation of hepatic stellate cells, extracellular matrix deposition, fibrosis, and eventually cirrhosis-like changes [209,210,211,212,213]. There, the counteractions occur with many agents, i.e., N-acetylcysteine. Because the model simultaneously reproduces oxidative stress, inflammation, endothelial dysfunction, microcirculatory disturbances, hepatocyte death, and progressive fibrogenesis, CCl_4_ intoxication has become a cornerstone model for evaluating hepatoprotective, antifibrotic, antioxidant, anti-inflammatory, angiogenic, and cytoprotective therapies. The studies establish N-acetylcysteine primarily as an antioxidant and glutathione-restoring agent [214,215,216,217]. Then, there are antifibrotic and anticirrhotic effects, and studies focus on fibrogenesis, cirrhosis progression, collagen accumulation, and chronic liver injury [218,219,220,221] as well as emphasize prevention of acute necrosis, hepatocyte death, and liver failure [215,217,222,223,224]. Additional papers compare N-acetylcysteine or are combined with other hepatoprotective agents [221,222,225].

Unlike the studies of Simko and colleagues [171], ursodeoxycholic acid is effective against CCl_4_-liver lesions [226,227,228,229].

Corticosteroids are also effective, including corticosteroids and anti-inflammatory steroid therapy [230,231,232,233], antioxidant steroid precursor dehydroepiandrosterone in particular [234,235] and endogenous glucocorticoids and hormonal modulation [236]. Notably, there was a demonstration of synergistic effects between prednisolone and phospholipid-containing hepatoprotectors [230].

Resveratrol is also effective. These would include anti-fibrotic signaling and hepatic stellate cell regulation [237,238,239], vascular/endothelial protection and portal hypertension [240], anti-inflammatory and cytokine modulation [239,241,242], antioxidant and hepatoprotective effects [242,243,244], drug delivery and formulation studies [241], fibrosis-associated signaling pathways [237,238,241], and computational/quantitative assessment studies [245].

Curcumin healing evidence is also complex. Specifically, it includes classical antioxidant and cytoprotective hepatoprotection [246,247,248,249,250,251,252] and antifibrotic effects through hepatic stellate cell (HSC) inhibition and apoptosis [253,254,255,256,257] and anti-inflammatory and immunomodulatory actions [258,259,260,261,262]. Angiogenesis and sinusoidal remodeling occur as a reduction of angiogenesis, sinusoidal capillarization, and vascular remodeling during fibrosis [263,264]. Molecular signaling pathways in fibrosis were also postulated [265,266]. These are also intended to improve bioavailability and hepatic targeting, as well as enhance antifibrotic efficacy [267,268,269,270].

Sildenafil is also effective [271,272,273].

Melatonin effectiveness includes several important points. The first point includes cirrhosis and antifibrotic mechanisms [274,275,276,277,278,279,280,281,282], then, oxidative stress and hepatoprotection [122,283,284,285,286,287,288,289,290,291,292,293], as well as mitochondrial protection and cellular stress responses [294,295,296]. There are also combination therapies and novel delivery approaches [297,298,299,300]. Finally, the studies include extrahepatic effects and functional outcomes [301,302,303,304].

Simvastatin includes studies primarily evaluating antifibrotic effects and fibrosis regression [305,306], portal hypertension, endothelial dysfunction, and hepatic hemodynamics [124,307,308]. Furthermore, there are studies investigating mitochondrial respiratory chain alterations and their correction by simvastatin [309,310] and steatohepatitis and metabolic liver injury [311].

Prostacyclin or prostacyclin analogs were shown as therapeutic interventions against CCl_4_-induced liver injury, endothelial damage, or hepatic necrosis [312,313,314,315,316]. This includes hepatic microcirculation, endothelial function, vascular signaling [317], arachidonic acid metabolism, and eicosanoid-mediated hepatoprotection [318].

For coenzyme Q10 supplementation and hepatoprotection against CCl_4_ toxicity, studies directly evaluated exogenous CoQ/ubiquinone as a protective therapy [29,132,133,319,320,321], together with other antioxidant therapies [132,133,322,323].

The strongest directly confirmed VEGF + CCl_4_ gene delivery evidence is adenoviral VEGF or modified VEGF (CBD-VEGF) in CCl_4_ fibrosis models. VEGF plasmid/viral delivery improved liver repair and microcirculation (multiple rodent studies). Some VEGF gene therapy studies are indirect (angiogenic pathway/MSC-mediated VEGF upregulation) rather than direct VEGF plasmid injection [324,325,326,327].

There are also stem cell therapies with VEGF activation improving CC_4_ liver lesions [328,329,330].

The studies that directly demonstrated beneficial effects of L-arginine (or L-arginine-based interventions) in CCl_4_-induced liver injury/cirrhosis models are relatively limited. Several papers investigated NO signaling or arginine metabolism mechanistically, but only a subset showed therapeutic benefit [331,332,333,334,335]. There are only a few studies that directly tested NOS inhibitors (NO blockers). They demonstrated worsening of liver injury or fibrosis [334,336,337]. Other studies did not support aggravation of liver lesions (improvement in renal function; study addressed circulatory abnormalities rather than liver injury) [338,339]. Thus, assuming CCl_4_ hepatotoxicity as a distinctive cytoprotective background that should emphasize cytoprotection commonality for all of the given agents, the sharing includes more agents than in the paracetamol liver lesions. This includes, as previously in paracetamol liver lesions, N-acetylcysteine, resveratrol, curcumin, melatonin, coenzyme Q10, corticosteroids, and sildenafil. The others, i.e., prostacyclin analogs, simvastatin, ursodeoxycholic acid, and experimental delivery of VEGF genes, were also in the loop. Similarly, these agents were used in all regimens. Pretreatment includes N-acetylcysteine [215], corticosteroids [234,235], resveratrol [243,244], curcumin [258,259,267,268], sildenafil [273], melatonin [293], and coenzyme Q10 [29,132,319,320,321]. Cotreatment includes N-acetylcysteine [214,218,219,220,221], ursodeoxycholic acid [227], corticosteroids [230,231,233], resveratrol [238,239,241,242], curcumin [257,258,259,260,261,262,263,264,266,267,268,269], sildenafil [272], melatonin [122,275,276,277,278,279,280,284,287,288,289,293,296,297,300,302], simvastatin [309,311], prostacyclin [313,316], and coenzyme Q10 [133]. Posttreatment includes N-acetylcysteine [222,223], ursodeoxycholic acid [226,228,229], resveratrol [237,240,244], curcumin [262,265,270], sildenafil [271], melatonin [274,281,282,286,290,291,292,294,298,299,301,304], simvastatin [305,306,307], and VEGF gene delivery [324,326,327], as well as stem cell therapies with VEGF activation [328,329,330]. Both pretreatment and posttreatment conditions include N-acetylcysteine [216,217,224], melatonin [283,285], and prostacyclin [312].

On the other hand, the CCl_4_ hepatotoxicity as a distinctive cytoprotective background may be markedly wider. This should be seen with an extensive number of other agents belonging to distinctive classes that counteract carbon tetrachloride hepatotoxicity in animal settings (Table 4).

On the other hand, the CCl_4_ hepatotoxicity as a distinctive cytoprotective background may be markedly wider. This includes extrahepatic protection, for instance, with N-acetylcysteine [215,217,222,223,224,362], melatonin [278,286,288,290,291,363,364] (liver oxidative stress, mitochondrial ROS regulation [278,286,288,290,291,363,364], extrahepatic: brain function + neuromotor recovery (gut–liver–brain axis) [302,365]), curcumin [253,256,258,260,263,264,267] (fibrosis reversal; systemic hepatoprotection [260,263], anti-angiogenic vascular remodeling [258,264,267]), resveratrol [237,238,239,240,244], and simvastatin [124,306,307,309,311].

Thus, carbon tetrachloride studies are not limited to liver injury alone as a model of acute liver failure but as a multiorgan disorder. Vice versa, considering the multitude of organs involved and multitude of the agents of different classes implicated in the beneficial effects, counteraction of the carbon tetrachloride occurs as a cytoprotection background transmitted to other organs therapeutically by the same (cytoprotection) agent application. As a follow-up, there is multiorgan protection, simultaneous protection of liver and extrahepatic organs that quite commonly occurs with the agents that protect against carbon tetrachloride-induced liver injury.

On the other hand, these points seem not to be appreciated in the clinic, although basic studies and noted effects of the implicated agents closely approached antidote-based hepatoprotection (N-acetylcysteine/acetaminophen) and multifactorial cytoprotection (CCl_4_ and other complex liver injury models).

Unlike, so far, the only established clinical antidote for acetaminophen poisoning [135], no direct antifibrotic drug was fully validated yet.

#### BPC 157/CCl_4_

BPC 157/CCl_4_ relation [37] should be perceived in the light of the presented findings with all of the mentioned agents. In the carbon tetrachloride studies, BPC 157 (10 µg/kg–10 ng/kg b.w. as a regular regimen also in paracetamol studies [36], given intraperitoneally and intragastrically [37]), was combined with the equal effect against liver lesions induced by ligation of the bile duct and hepatic artery and counteraction of the restraint-stress-induced liver lesions. This may be relevant when discussing the robustness and translational significance of the observed protective effects [37]. Although this was not specifically investigated in BPC 157 carbon tetrachloride liver lesions studies, given that protection against CCl_4_-induced hepatic injury in experimental animals reflects modulation of conserved pathways of oxidative stress, inflammation, stellate cell activation, and sinusoidal endothelial dysfunction, these were later demonstrated in the subsequent studies. Therefore, that could imply BPC 157 carbon chloride evidence with circumstances that are also implicated in human liver diseases, such as toxic hepatitis, MASLD/MASH, and liver fibrosis/cirrhosis.

### 2.3. Bile Duct Ligation Model of Liver Injury: Significance and Relevance to Cytoprotection

Bile duct ligation is one of the most widely used experimental models of cholestatic liver injury, secondary biliary fibrosis, portal hypertension, and progressive liver failure. Following surgical obstruction of the common bile duct, bile flow from the liver to the intestine is interrupted, resulting in intrahepatic accumulation of toxic bile acids, cholestasis, oxidative stress, inflammation, hepatocyte and cholangiocyte injury, activation of hepatic stellate cells, fibrosis, cirrhosis-like remodeling, and portal hypertension [365,366,367,368]. Unlike acetaminophen intoxication, which primarily models acute toxic hepatocellular necrosis, or CCl_4_ administration, which mainly reproduces toxic oxidative injury and fibrosis, the bile duct ligation model reproduces the complex pathophysiology of chronic cholestatic liver diseases [369,370], including cholestasis, bile acid toxicity, inflammatory activation, oxidative stress, endothelial dysfunction, sinusoidal remodeling, fibrosis, portal hypertension, hepatic encephalopathy, and multiorgan complications in advanced stages.

Therefore, bile duct ligation represents a particularly valuable model for evaluating therapies aimed at preserving liver structure and function under conditions of impaired bile flow and progressive vascular and fibrotic remodeling.

The significance for the cytoprotection concept should be particularly emphasized. From the cytoprotection perspective, the bile duct ligation model is especially important because it simultaneously involves several major targets of cytoprotective therapy [370]. These are preservation of hepatocyte viability, protection of cholangiocytes, [370,371,372], maintenance of endothelial integrity, and improvement of hepatic microcirculation [373,374,375]. Likewise, these are modulation of nitric oxide pathways, attenuation of oxidative stress, suppression of inflammation [374,375], and limitation of fibrosis progression [370,371,376]. Finally, there is also counteraction of portal hypertension [373,374]. Thus, successful therapy in the bile duct ligation model may indicate broader cytoprotective activity extending beyond simple hepatocyte protection.

These include the mentioned agents’ shared effectiveness. N-acetylcysteine was effective [377,378,379,380,381,382,383]. Resveratrol was effective [384,385,386,387]. Curcumin was effective [256,388,389]. Melatonin was also shown to be effective [390,391,392,393,394,395,396,397]. Sildenafil was effective as well [115,398,399,400], and ursodeoxycholic acid was effective [401,402,403,404,405,406,407]. Simvastatin was also effective [408]. Corticosteroids were effective [409,410,411,412,413]. Prostacyclin and prostaglandin E analogues were effective [414,415,416,417,418]. There are also studies showing the effectiveness of VEGF gene delivery [419,420] and stem cell therapy with VEGF activation [421]. Notably, the effectiveness of these agents’ application includes pretreatment, cotreatment, and posttreatment. Pretreatment includes ursodeoxycholic acid [400,405], simvastatin [408], and prostacyclin [417,418]. Cotreatment includes N-acetylcysteine [380,382,383], resveratrol [385,386,387], curcumin [289], melatonin [393,396,397], sildenafil [397,398], and ursodeoxycholic acid [401,402,405]. Posttreatment includes N-acetylcysteine [377,378,379,381], resveratrol [384,385,386,387], curcumin [256,288,289], melatonin [390,391,392,394,395], sildenafil [115,396], ursodeoxycholic acid [402,403,404,407], corticosteroids [409,410,411,412], and prostacyclin [414,415].

For the L-arginine, however, the Mayoral et al., 1999 study [422] showed that while bile duct ligation alone increases fibrosis, collagen deposition, and iNOS expression, and NO production, L-arginine further increases NO production and inducible NOS activity. This leads to aggravation, higher hepatic collagen content, and greater fibrosis area. An aggravating effect has also L-NAME, which may worsen the signs of cholestasis and cellular injury and biochemical injury markers [422]. Thus, although, in general, L-arginine and L-NAME are distinct compounds with opposing effects on the NO system, this study [422] indicates an additional L-arginine/L-NAME parallel action, which also quite commonly occurs with NO-agents effects, and was recently reviewed [10].

Broader cytoprotective activity appears based on the agents’ effectiveness in bile duct ligation-induced liver injury since they also ameliorate extrahepatic lesions (kidney, brain, lung, stomach, vascular system), supporting the concept of systemic protection. There are N-acetylcysteine [377,379], resveratrol [386], melatonin [393,396,397], ursodeoxycholic acid [405,407], sildenafil [398], and prostaglandin E1 analogues [415]. Neurological manifestations of bile duct ligation were improved by steroid precursors [409,412].

On the other hand, it is interesting that considering the inherent combination of bile-duct-ligation-induced cirrhosis and portal hypertension, only a subset actually investigated portal hypertension or portal pressure as a primary endpoint in the bile duct ligation rat model. These are ursodeoxycholic acid [404], sildenafil [115], and N-acetylcysteine [404]. As pointed out, many studies examined fibrosis, oxidative stress, liver histology, or microcirculation without directly measuring portal pressure.

Finally, the additional potential of bile-duct-ligation-induced liver lesions should be seen with an extensive number of other agents belonging to distinctive classes that counteract bile-duct-ligation-induced hepatotoxicity in animal settings (Table 5).

#### BPC 157/Bile-Duct-Ligation-Induced Liver Lesion

The bile-duct-ligation-induced liver lesions study verified particularity of the BPC 157 efficacy. BPC 157 medication (10 µg/kg, 10 ng/kg) per-orally or intraperitoneally is a robust protocol, providing similar effectiveness of two ways of administration in the same model. On the other hand, it seems to be quite complete, given both liver cirrhosis prevention, starting with therapy immediately after surgery, and already established lesion reversal, providing therapy at week 4 onward [38]. Local bath BPC 157 (10 µg/kg) with assessed immediate normalization of portal hypertension was given immediately after establishing portal hypertension values at 4, 6, and 8 weeks. BPC 157 therapy markedly abated jaundice, snout, ears, paws, and yellow abdominal tegmentum in controls since the 4th week; ascites, nodular, steatotic liver with large dilatation of the main bile duct; increased liver and/or cyst weight; and decreased body weight. BPC 157 counteracts the piecemeal necrosis, focal lytic necrosis, apoptosis and focal inflammation, disturbed cell proliferation (Ki-67-staining), cytoskeletal structure in the hepatic stellate cell (α-SMA staining), and collagen presentation (Mallory staining). Likewise, counteraction includes increased AST, ALT, GGT, ALP, total bilirubin, direct and indirect and decreased albumin serum levels. As the end result, normalized MDA- and NO-tissue values appear, next to Western blot of NOS2 and NOS3 in the liver tissue and decreased IL-6, TNF-α, IL-1β levels in liver tissue. Finally, although portal hypertension is sustained in bile-duct-ligated rats, with BPC 157 therapy, portal hypertension in bile-duct-ligated rats is either not even developed or rapidly abated, depending on the given BPC 157 regimen. Thus, BPC 157 may counteract liver fibrosis and portal hypertension.

This particular recovering effect should likely be suggested, given a short summary of all the important points. The reported effects included reduction of portal hypertension, improvement of liver biochemical abnormalities (AST, ALT, GGT, ALP, bilirubin, albumin), attenuation of fibrosis-related changes, reduction of inflammatory changes, improvement of histological liver lesions, and changes in markers related to hepatic stellate cell activation (α-SMA), collagen deposition, apoptosis, and proliferation (Ki-67) [38].

### 2.4. Alcohol-Induced Liver Lesions in Rats and Mice and Their Possible Significance

From a general cytoprotection viewpoint provided in funding studies [1,2,3,4,5,6,7,8,9], alcohol as a prototype noxious challenge, alcohol-induced liver lesions may have a particular indicative significance, more than any other model previously mentioned (i.e., acetaminophen, CCl4, bile duct ligation). Notably, the history of cytoprotection begins with Robert’s original experiments with intragastric administration of absolute ethanol and prostaglandins, extensive gastric mucosal necrosis vs. the general preservation of cellular integrity and tissue resistance via cytoprotection agent application against direct noxious injury. Conceptually, prostaglandins also prevented the liver lesions [439], and therefore, in cytoprotection funding studies, alcohol-induced liver injury represents an extension of the same general experimental principle to a second major target organ [1,2,3,4,5,6,7,8,9,439], in principle, ethanol-induced gastric cytoprotection→organoprotection→ethanol-induced liver cytoprotection. Ethanol exposure in both settings involves direct cellular injury, oxidative stress, microcirculatory and endothelial disturbances, inflammation, and, particularly during chronic exposure, progressive structural damage.

In general basic terms, alcohol-induced liver injury in rats and mice closely resembles several key features of human alcohol-associated liver disease. The most common lesions include hepatic steatosis, steatohepatitis, fibrosis, and in some models, cirrhosis. Ethanol metabolism generates acetaldehyde and reactive oxygen species, leading to oxidative stress, inflammation, hepatocyte injury, and impaired liver regeneration. Alcohol-induced liver lesions are highly relevant because they represent a progressive pathological continuum that can evolve from simple steatosis to steatohepatitis, fibrosis, cirrhosis, portal hypertension, and ultimately hepatocellular carcinoma [440,441,442]. Finally, the resulting inflammatory-fibrotic microenvironment promotes activation of hepatic stellate cells and accelerates progression toward cirrhosis and hepatocellular carcinoma. Therefore, therapies capable of simultaneously reducing oxidative stress, inflammation, pathological proliferation, and fibrosis may have particular value in preventing the fibrosis–cirrhosis–carcinoma sequence characteristic of advanced alcohol-associated liver disease [443].

On the other hand, in the alcohol-induced liver lesions, the effects of drugs seem to be more limited than in other models. There is evidence of the effectiveness of N-acetylcysteine [444,445]. Interestingly, N-acetylcysteine pretreatment protected against acute ethanol-induced liver injury, reducing oxidative stress, glutathione depletion, and TNF-α expression, whereas post-treatment 4 h after ethanol aggravated liver injury in a dose-dependent manner [445]. Effective treatments were curcumin [446], resveratrol [447], melatonin [448,449,450], and coenzyme Q10 [451]. Ursodeoxycholic acid can act beyond its established cholestatic indications and influence alcohol-associated hepatic injury [452,453]. PGE_1_ attenuates alcohol-enhanced liver injury [454]. Illustratively, N-acetylcysteine was administered as part of the enteral nutrition regimen during the ethanol-feeding period [444]. Likewise, curcumin was administered during the ethanol-feeding period [446]. Resveratrol was administered concurrently with chronic ethanol exposure throughout the experimental period [447]. Melatonin was given during alcohol challenge [448,449,450]. Coenzyme Q10 was given during alcohol challenge over 30 days [451]. Ursodeoxycholic acid was administered for the same 30 days [452,453]. Moreover, 30 µg/kg of a prostaglandin E1 (PGE1) analogue 24 h before endotoxin administration represents a clear pretreatment protocol [454].

Notably, as an additional part of the limitations, they are all [444,445,446,447,448,449,450,451,452,453,454] predominantly preventive/prophylactic or concomitant exposure studies. There is no convincing posttreatment/reversal model in which the agent was started after alcohol-induced liver lesions were already established and then shown to reverse those lesions. Likewise, there is no demonstration of concomitant counteraction of alcohol-induced portal hypertension. Considering corticosteroids, prednisolone was tested in several experimental liver-injury models, including chronic + binge ethanol feeding. Importantly, prednisolone enhanced ethanol-induced liver injury rather than protecting against it [455]. The inhibition of liver regeneration and macrophage/neutrophil phagocytosis was a possible explanation [456].

Considering gastric/liver alcohol lesions and their counteraction, specifically alcohol-induced gastric lesions, as an original supportive argument, these can be clearly stated for N-acetylcysteine [457,458,459], curcumin [460,461], resveratrol [462], melatonin [463,464,465], and PGE_1_ [466]. On the other hand, coenzyme Q10 failed [467]. Ursodeoxycholic acid was not tested, but tauroursodeoxycholic acid was protective [468]. Notably, like with the inconsistencies in alcohol-induced liver lesions [445], there are similar points with N-acetylcysteine (no effect, or dual effects) [458,459]. Finally, unlike the effect on liver lesions, prednisolone is beneficial with alcohol-induced gastric lesions [469].

Considering the counteraction of alcohol-induced liver lesions along with alcohol-induced drunkenness, withdrawal, these were not directly investigated. Most evidence was noted with N-acetylcysteine after cessation of chronic alcohol administration [470], ethanol dependence, self-administration, motivation, seeking and relapse [471], or acute post-ethanol behavioral/neurological consequences (hangover) [472]. There is also evidence for curcumin [473], resveratrol [474], and melatonin [475,476].

#### BPC 157 and Alcohol-Induced Liver Lesions

The evidence collected from BPC 157 studies suggests that an agent capable of counteracting both gastric and hepatic alcohol-induced injury may achieve a broader cytoprotective rescuing action in the whole alcohol issue rather than an effect restricted to one tissue or one particular lesion [10]. Indicatively, portal hypertension and liver lesions in chronically alcohol-drinking rats were prevented and reversed by stable gastric pentadecapeptide BPC 157. In this model, rats received alcohol chronically for three months [39]. BPC 157 was administered either throughout the alcohol-drinking period or during the final month, after two months of continuing alcohol consumption, and after the pathological condition had developed. The reported endpoints were directly measured portal pressure, liver lesions, steatosis, hepatocyte and hepatocyte nuclear alterations, and increased liver weight. There, BPC 157 reduced the elevated portal pressure to values not different from healthy rats and attenuated or eliminated several associated hepatic disturbances. Importantly, the therapeutic protocol was applied after prolonged alcohol exposure and was interpreted as reversing already established portal hypertension and associated liver injury [39].

Thus, as a major difference, unlike the predominantly prophylactic or concomitant protocols, without portal hypertension assessment [444,445,446,447,448,449,450,451,452,453,454], reported for several standard agents in experimental alcohol-induced liver injury, BPC 157 was also investigated in chronically alcohol-drinking rats under a delayed-treatment protocol, in which treatment during the final month of continued alcohol exposure counteracted established portal hypertension and associated liver disturbances. Similar beneficial effects, obtained by two routes of administration (intraperitoneal/intragastric) and using comparative agents (propranolol, ranitidine), also contribute to the robustness of the findings [39].

Moreover, the additional study of acute absolute alcohol intragastric application reinterprets the classical intragastric 96% ethanol model [40]. The consistent evidence showed that BPC 157 administration, in addition to counteraction of severe alcohol lesions in the stomach, simultaneously counteracted severe liver lesions and congestion and, most importantly, portal hypertension as well. These beneficial effects were along with counteraction of other blood pressure disturbances, such as intracranial hypertension, caval hypertension, and aortal hypotension, and counteraction of widespread thrombosis, and severe lesions in other organs (hemorrhage), brain, heart, lung and kidney. Counteraction of this severe vascular and multiorgan failure was explained by a prompt activation of the collateral vascular pathways (i.e., azygos vein direct blood flow recovery) and reversal of the already advanced Virchow triad circumstances.

Finally, BPC 157 may have a beneficial effect against acute alcohol intoxication or withdrawal following chronic consumption [477]. In mice, BPC 157 (10 pg intraperitoneally, 10 ng and 10 µg, intraperitoneally or intragastrically) strongly opposed acute alcohol (4 g/kg intraperitoneally) intoxication (i.e., quickly produced and sustained anesthesia, hypothermia, increased ethanol blood values, 25% fatality, 90-min assessment period) given before or after ethanol. Likewise, when given after abrupt cessation of ethanol (at 0 or 3 or 7 h withdrawal time), it attenuated withdrawal (assessed through 24 h) after 20—alcohol drinking (7.6 g/kg) through 13 days, with provocation on the 14th day. Thus, it seems that BPC 157 consistently acts as an ethanol antagonist. As pointed out, it consistently provides benefit for the otherwise opposite alcohol events elicited by acute (i.e., acute ethanol generally enhances inhibitory signaling, particularly GABAergic 742 activity, and suppresses important components of excitatory glutamatergic signaling) or chronic alcohol disturbances (compensatory enhancement of excitatory glutamatergic mechanisms and alterations or reductions in inhibitory GABAergic function) [477].

Thus, considering the models investigated (acetaminophen, CCl_4_, bile duct ligation, and now alcohol-induced liver lesions), it seems that BPC 157’s efficacy in particular extends over the effects shown with comparative hepatoprotective agents. Indicatively, in alcohol injury, it behaves somewhat differently from a conventional single-target ethanol antagonist: its effects appear to extend across opposite or sequential states of ethanol disturbance rather than being limited to prevention of one particular lesion.

### 2.5. Further Evidence of Occlusion/Occlusion-Like Syndrome, Including Alcohol-Induced Injury, Hepatic Ischemia/Reperfusion, Pringle Maneuver, Budd–Chiari Syndrome, Inferior Caval Vein and Superior Mesenteric Vessel Occlusion, Abdominal Compartment Syndrome, Pancreatitis, Bile Duct Obstruction, Toxic and Drug-Induced Vascular Failure, Embolization, Systemic Ischemia/Reperfusion, and Radiation-Induced Liver Injury

The evidence goes across diverse rat models. These include alcohol-induced injury [40], hepatic ischemia/reperfusion, Pringle maneuver [42], Budd–Chiari syndrome [41], inferior caval vein [41,43] and superior mesenteric vessel occlusion [44,45,46], central vessel (occlusion of the superior sagittal sinus) occlusion [47], adrenalectomy [51], abdominal compartment syndrome [48,49], pancreatitis, bile duct obstruction [50], toxic and drug-induced vascular failure [52,53,54,55,56,57], embolization [57], systemic ischemia/reperfusion [42,48,49,58], and radiation-induced liver injury [59]. As a shared effect [40,41,42,43,44,45,46,47,48,49,50,51,52,53,54,55,56,57,58,59], BPC 157 consistently counteracted hepatic and extrahepatic injury associated with vascular failure, venous congestion, ischemia, reperfusion, thrombosis, and disturbed pressure relationships. The most reproducible finding was the rapid restoration of vascular function through activation of collateral pathways (confirmed also by angiography and venography). In particular, BPC 157 recruited alternative portal, mesenteric, portocaval, azygos/hemiazygos, and caval venous routes, thereby improving venous drainage and/or restoring arterial inflow when major vessels were obstructed. Consequently, the accompanying portal and caval hypertension and intracranial hypertension and aortic hypotension were eliminated and/or markedly attenuated, while thrombosis, venous congestion, ascites, and multiorgan dysfunction were reduced.

There, as a particular emphasis, along with previous counteraction of portal hypertension described in bile-duct-ligation rats and alcohol-induced liver lesions [38,39,40], this evidence strongly suggests that as a consequence, BPC 157 has a broader effect and equally counteracts prehepatic, hepatic, and posthepatic portal hypertension [40,41,42,43,44,45,46,47,48,49,50,51,52,53,54,55,56,57].

Along with this, in the liver, these vascular effects were associated with less hepatic congestion, dilation of central veins and terminal portal venules, sinusoidal enlargement, hepatocellular injury, oxidative stress, and increases in serum liver enzymes. Importantly, these effects were observed both during the initial ischemic/occlusive period and after decompression or reperfusion, indicating protection against both vascular obstruction and the subsequent reperfusion injury [40,41,42,43,44,45,46,47,48,49,50,51,52,53,54,55,56,57,58,59].

The findings also indicate that the hepatic effects cannot be attributed exclusively to restoration of blood flow. In models of radiation-induced liver injury and systemic ischemia/reperfusion, BPC 157 directly reduced hepatocellular injury, apoptosis, lipid accumulation, and oxidative disturbances; in radiation injury, the protective effect was linked to KLF4 upregulation [59]. Thus, the experimental evidence supports complementary vascular and cellular components of BPC 157 cytoprotection. The vascular component appears particularly prominent in models in which liver injury develops together with portal/caval hypertension and systemic circulatory failure, whereas the direct cellular component becomes more evident in models of radiation or distant-organ ischemic injury.

Taken together, these studies suggest a common sequence: vascular obstruction or injury → impaired collateral circulation → portal/caval congestion → portal hypertension and aortic hypotension → hepatic congestion and ischemia → thrombosis, oxidative stress and multiorgan injury [40,41,42,43,44,45,46,47,48,49,50,51,52,53,54,55,56,57,58,59]. BPC 157 acts in the opposite direction: BPC 157 → collateral vascular recruitment and restoration of vascular flow → reduction of portal/caval hypertension → improved hepatic and systemic perfusion → reduction of congestion and thrombosis → attenuation of oxidative/reperfusion injury → preservation of liver and other organs [40,41,42,43,44,45,46,47,48,49,50,51,52,53,54,55,56,57,58,59].

In summary, this interpretation is especially supported by the Pringle maneuver and Budd–Chiari models [41,42], in which the restoration of collateral circulation occurred together with normalization of portal/caval hemodynamics and marked improvement of hepatic pathology. The central [47] and peripheral vessel occlusion, superior sagittal sinus, inferior caval vein, superior mesenteric vessel occlusion [41,43,44,45,46], and abdominal compartment syndrome studies [48,49] further demonstrate that the same response can operate when portal hypertension arises from central and inferior caval vein or mesenteric venous obstruction or generalized intra-abdominal vascular compression. The alcohol, pancreatitis, bile duct obstruction, toxic-drug, adrenalectomy, embolization, and systemic ischemia models [51,52,53,54,55,56,57,58] broaden the phenomenon, suggesting that it is not restricted to a single anatomical lesion. In particular, BPC 157 counteracts experimentally induced portal hypertension, reducing elevated portal pressure and improving the associated hepatic and vascular disturbances, with evidence for both prevention of its development and reversal of established portal hypertension [40,41,42,43,44,45,46,47,48,49,50,51,52,53,54,55,56,57].

Finally, in addition, there are BPC 157 studies where the primary insult is surgical loss of 70% of liver mass [478,479,480]. They can be important because they demonstrate BPC 157 in a model that differs fundamentally from acetaminophen, CCl_4_, bile duct ligation, alcohol, or other chemically induced liver injuries. BPC 157 consistently improved the recovery of the remnant liver, as evidenced by better restoration of liver mass and volume, improved biochemical indices of hepatic injury and function, and more favorable histological and regenerative findings. Thus, there were limitations in secondary hepatocellular injury, stimulation or normalization of regeneration, and prevention of steatosis, necrosis, and fibrosis, and these effects were observed with both µg/kg and ng/kg doses and after intraperitoneal or oral administration. Additionally, these effects were combined with maintenance of the appropriate NO-system function (counteraction of L-NAME-aggravation) [478,479,480].

## 3. Final Remarks

Notably, the specific points discussed before should be again emphasized as, on the other hand, they might still indicate general concern. The credibility of all findings in the BPC 157 therapy story is tightly dependent on the relevance and general accuracy of the grand concepts of Robert and Szabo cytoprotection/organoprotection [1,2,3,4,5,6,7,8,9]. Thus, the issue can be cytoprotection as a unifying conceptual therapeutic effect, a point specifically explained in our recent reviews [30,31,99,100,481]. When, conceptually, the essential cytoprotection ↔ hepatoprotection background is omitted, complaints may arise. Without consideration of such cytoprotection ↔ hepatoprotection background, uncertainty favors a lack of proper balance, suspecting significant focus on BPC 157 and its particular broad and restorative capacity, claiming research groups as similar and, thereby, lacking independent replication.

Contrarily, for the hepatoprotection from a cytoprotective viewpoint, various agents have shown essential cytoprotective properties (i.e., specifically, maintaining and/or reestablishing hepatocellular, endothelial, and tissue integrity) (see, i.e., Section 2.1, Section 2.2, Section 2.3 and Section 2.4). This illustrates that cytoprotection, also hepatoprotection, is not a distinct pharmacological class but rather a shared therapeutic property across diverse drug groups and, consequently, their broad and restorative capacity. There, the stable gastric pentadecapeptide BPC 157 has emerged as a particularly relevant cytoprotective therapy. There, the basis is the cumulative evidence obtained from all of these analyzed comparative agents’ activities on classic liver lesions models. Consequently, this can underline its particular broad and restorative capacity and gradual distinction from the other studied standard agents (i.e., capability to both prevent and reverse already established prehepatic, hepatic, and posthepatic portal hypertension [38,39,40,41,42,43,44,45,46,47,48,49,50,51,52,53,54,55,56,57]). Furthermore, in BPC 157 review studies, mentioned before, using cytoprotection as a unifying strategy [30,31,99,100,481] in settings outside the liver topic, a similar conclusive approach was used. Purposefully, its particular broad and restorative capacity as a shared cytoprotective point is based on the cumulative evidence collected in these reviews [30,31,99,100,481] from all of the analyzed comparative agents’ activities on classic corresponding lesion models. Therefore, the breadth of BPC 157 is not asserted in isolation; it is derived from the cumulative comparison of BPC 157 with the effects of the other agents across classical liver-lesion models. As a result, this conclusion in the liver lesions (broad and restorative capacity of BPC 157 therapy) is in accordance with previous findings supporting each other [30,31,99,100,481].

In practical terms of recognition, even before this particular moment of rapidly increased interest (i.e., FDA advisory committee), several reviews authorized by other groups have fully recognized BPC 157 significance [482,483,484,485,486,487,488,489,490]. Furthermore, in addition to our reviewed findings (Section 2.1, Section 2.2, Section 2.3, Section 2.4 and Section 2.5) and findings obtained in collaboration with other labs [80,81,491], there is continuous support from additional studies carried independently by various other groups [82,83,84,85,86,87,89,90,492,493,494,495,496,497,498,499,500,501,502,503,504,505,506,507]. Consistently, independent replications have demonstrated that BPC 157 regularly exerts a significant protective effect. Quite recently, additional reviews have appeared from additional research groups, some instructive for further application in therapy [508,509,510,511,512,513,514,515,516]. Notably, such recognition should not be equated with established clinical efficacy.

Still, taking all these things together, there is always a question of the practical point of the applicable results and likely translation possibilities. Nevertheless, studies have systematically evaluated the compound’s efficacy following oral, intraperitoneal, and intragastric administration, often within the same pathological model. Moreover, there is often increased severity in the pathological models used. Important for liver lesions as a part of acute abdominal compartment syndrome ischemia/reperfusion, BPC 157 studies considered the effects during very high intra-abdominal pressure values, 25–50 mmHg, while regular standard agent assessment considers only lower intra-abdominal pressure values, those below 20 mmHg [481]. Therefore, hopefully considering BPC 157’s µg-ng dose range, applied alone without carrier addition, and its application, including via the per oral route, clinical evidence that should be further obtained, and toxicology without LD1, we can ascertain its practical applicability. Indeed, through in situ hybridization and immunostaining, BPC 157 was found in humans, in both adult and fetal tissues, gastrointestinal mucosa, lung bronchial epithelium, the epidermal layer of the skin, and kidney glomeruli [10,517], and therefore may have a regulatory role.

## 4. Conclusions

The cytoprotection concept provides a broad framework for understanding liver injury and its treatment, in which preservation of cellular integrity is closely connected with maintenance of endothelial function, microcirculation, oxidative and inflammatory balance, and adequate vascular compensation. The experimental evidence reviewed here indicates that liver injury is not restricted to hepatocyte damage but involves interconnected hepatic and extrahepatic vascular disturbances that may determine the severity and reversibility of organ injury. Accordingly, effective cytoprotective therapy may require simultaneous protection and restoration of these interconnected systems.

Standard pharmacological and biological agents demonstrate that individual components of this process can be therapeutically modified. N-acetylcysteine, ursodeoxycholic acid, corticosteroids, antioxidants, agents affecting the NO-system, growth-factor-based approaches, and cellular therapies have shown beneficial effects in particular experimental models. Their effects, however, are frequently dependent on the type and stage of injury, the therapeutic window, and the dominant pathological mechanism.

Against this background (i.e., whatever animal study may serve as an indication, imitation, or limitation (i.e., only male rats used)), stable gastric pentadecapeptide BPC 157 represents a particularly broad experimental cytoprotective approach. Across different models, including alcohol-related injury, CCl_4_ and acetaminophen hepatotoxicity, cholestatic injury, ischemia-reperfusion, portal hypertension, and occlusion/occlusion-like syndromes, BPC 157 repeatedly demonstrated protective and restorative effects. The reported actions extend beyond hepatocytes to endothelial integrity, microvascular circulation, thrombosis, collateral vessel activation, NO-related mechanisms, angiogenic pathways, and recovery of other organs. The findings in portal hypertension are particularly relevant, suggesting that restoration of vascular patency and collateral circulation may contribute substantially to the reduction of portal and systemic consequences. This includes prehepatic, hepatic, and posthepatic portal hypertension counteraction. It is always combined with the similar counteracting effect on caval and intracranial hypertension and aortal hypotension.

Overall, the available evidence supports the view that BPC 157 should be considered an experimental cytoprotective and vascular-restorative candidate rather than simply another hepatoprotective antioxidant. Its consistent activity against otherwise diverse injuries may reflect an ability to restore disturbed homeostasis at several interconnected levels. Nevertheless, the evidence remains predominantly preclinical, and further independent experimental and clinical studies are required to establish its pharmacological profile, safety, therapeutic efficacy, and applicability to human liver disease. The principal value of the present evidence is therefore to support cytoprotection as an integrated therapeutic concept and to identify BPC 157 as a promising candidate for further translational investigation, further independent replication, and phase II/III safety/efficacy data.

## Figures and Tables

**Table 2:** Representative Agent(s) Shown to Counteract Acetaminophen Hepatotoxicity.

Pharmacological Class	Representative Agent(s) Shown to Counteract Acetaminophen Hepatotoxicity	Representative References
**Thiol-containing antidotes and sulfur donors**	N-acetylcysteine, taurine, hypotaurine	[102,181]
**Histamine H2-receptor antagonists**	Cimetidine	[182,183,184]
**Alcohol dehydrogenase/CYP inhibitor antidotes**	Fomepizole (4-methylpyrazole)	[185]
**Anticonvulsants/CYP inhibitors**	Stiripentol	[186]
**Phosphodiesterase inhibitors**	Sildenafil (PDE5 inhibitor), Rolipram (PDE4 inhibitor) Aminophylline (PDE-non-specific)	[186,187]
**Leukotriene receptor antagonists**	Montelukast	[188]
**Melatonergic antidepressants**	Agomelatine	[189]
**α2-Adrenergic receptor agonists**	Dexmedetomidine	[190]
**Calcium channel blockers**	Amlodipine	[191]
**ACE inhibitors**	Lisinopril	[191]
**Xanthine oxidase inhibitors**	Allopurinol	[191]
**Growth factors and regenerative therapies**	G-CSF	[192]
**Endogenous peptide hormones**	Ghrelin	[193]
**Cytoprotective peptides**	BPC 157	[36]
**Polyphenolic antioxidants**	Curcumin, Resveratrol, Quercetin Epigallocatechin-3-gallate	[152,194]
**Carotenoid antioxidants**	Fucoxanthin	[195]
**Mitochondrial redox agents**	Leuco-methylene blue	[196]
**Phenolic antioxidants**	3,4-Methylenedioxyphenol	[197]
**Vitamin supplementation**	Folic acid	[198]
**Eicosanoids/prostaglandins**	Prostaglandin E2	[199]

**Table 3:** Representative Agent(s) Shown to Counteract Acetaminophen Hepatotoxicity and Induce Other Organ Protection.

Agent	Liver Protection	Other Organ Protection	Reference
**N-acetylcysteine**	Hepatoprotection	Nephroprotection, testicular protection	[153]
**Curcumin**	Hepatoprotection	Renal protection, testicular protection, hematological protection	[151,152,201]
**Folic acid**	Hepatoprotection	Nephroprotection	[198]
**Dexmedetomidine**	Hepatoprotection	Nephroprotection	[190]
**Montelukast**	Hepatoprotection	Known renoprotective and pulmonary protective effects in other oxidative injury models; APAP study focused on liver	[188]
**Coenzyme Q10**	Hepatoprotection	Mitochondrial protection in multiple tissues; systemic antioxidant effects	[160,162]
**Melatonin**	Hepatoprotection	Nephroprotection	[157,202]
**Resveratrol**	Hepatoprotection	Nephroprotection and broader organ protection through SIRT1/AMPK activation	[147,150]
**Ghrelin**	Hepatoprotection	Well-known protective effects in kidney, gut, heart, and CNS injury models	[193]
**Fucoxanthin**	Hepatoprotection	Potential systemic antioxidant effects, but APAP study evaluated mainly liver	[195]
**Thyme extract**	Hepatoprotection	Bone marrow protection and hematological improvement	[203]
**Hyperbaric oxygen + NAC**	Hepatoprotection	Reduced systemic inflammation and oxidative injury	[139]

**Table 4:** CCl_4_ Hepatoprotection—Pharmacological Classification Table.

Pharmacological Class	Representative Agent(s) Shown to Counteract CCl_4_ Hepatotoxicity	Representative References
**H_2_-receptor antagonists**	Cimetidine, Ranitidine	[340]
**H_2_-receptor antagonists (chemical toxicity model support)**	Cimetidine class compounds	[341]
**Flavonoids/polyphenols**	Quercetin	[342]
**Flavonoids/hepatoprotective phytochemicals**	Silymarin	[343]
**Amino acid derivatives/antioxidants**	Taurine, Hesperidin	[344]
**PPAR-γ agonists/metabolic modulators**	Pioglitazone, Cod liver oil	[345]
**Xanthine oxidase inhibitor**	Allopurinol	[346]
**Xanthine oxidase inhibitor (fibrosis** **reversal study)**	Allopurinol	[347]
**RAAS inhibitors (AT1 receptor** **blockers/ACE inhibition)**	Losartan	[348]
**RAAS inhibitors (ACEi/ARB class)**	Captopril, Candesartan	[349]
**Calcium channel blockers**	Nitrendipine, Nimodipine, Nisoldipine	[350]
**Endocrine growth factors**	IGF-I	[351]
**Growth hormone/cytokine therapy**	Growth hormone + IFN-γ	[352]
**Thyroid hormones**	Triiodothyronine (T3)	[353]
**Leukotriene receptor antagonists**	Montelukast	[354]
**AMPK activators/metabolic drugs**	Metformin	[355]
**Iron chelators**	Deferoxamine	[356]
**Metal ions/micronutrients**	Magnesium	[357]
**Selenium/antioxidants**	Selenium nanoparticles + plant extract	[358]
**Zinc oxide nanoparticles**	ZnO nanoparticles	[359]
**Mitochondrial** **modulators/nanoformulations**	Berberine nanoparticles	[360]
**Fat metabolism modulators (PPAR-α agonists)**	Clofibric acid	[361]

**Table 5:** Bile Duct Ligation (BDL) Hepatoprotection—Pharmacological Classification Table.

Pharmacological Class	Representative Agent(s) Shown to Counteract BDL-Induced Liver Injury	Representative References
**H_2_-receptor antagonists (historical evidence)**	Cimetidine, Ranitidine	[423]
**Flavonoids/polyphenols**	Quercetin	[424,425]
**Flavonolignan phytotherapy**	Silymarin	[426]
**S-adenosylmethionine derivatives/methyl donors**	S-Adenosyl-L-methionine (SAMe)	[427,428]
**RAAS inhibitors (AT1 receptor blockers)**	Losartan	[429]
**RAAS inhibitors (ACE inhibitors)**	Perindopril	[430]
**Endothelin receptor antagonists**	Bosentan	[431]
**Xanthine oxidase inhibitors**	Oxypurinol	[432]
**PPAR-γ agonists**	Pioglitazone	[433]
**FXR agonists/bile acid signaling modulators**	Obeticholic acid (INT-747)	[434]
**NO pathway modulators**	V-PYRRO/NO	[435]
**Cannabinoid system modulators**	SR141716A (Rimonabant)	[436]
**Antioxidant vitamins**	Vitamin E (α-Tocopherol)	[437]
**HMG-CoA reductase inhibitors** **(excluding simvastatin)**	Lovastatin	[438]
**Antifibrotic methylxanthines**	Pentoxifylline	[411]

## Data Availability

No new data were created or analyzed in this study. Data sharing is not applicable to this article.
